# PSMA PET/CT-Guided Multimodal Therapy for Pelvic Lymph Node Positive and De Novo Low-Volume Metastatic Prostate Cancer: A Gulf Region Single-Institution Experience

**DOI:** 10.3390/diseases14070232

**Published:** 2026-06-28

**Authors:** Nadeem Pervez, Benazir Mir Khan, Sharjeel Usmani, Hasan Al-Sayegh, Iqbal Al Amri, Mahmoud Alfishawy, Sercan Yilmaz, Sulaiman Al Saadi, Munjid Al Harthy, Javeria Ahmed, Zahid Almandhari

**Affiliations:** 1 Department of Internal Medicine, College of Medicine and Health Sciences, United Arab Emirates University, Abu Dhabi P.O. Box 15551, United Arab Emirates; 2 Radiation Oncology, Saskatoon Cancer Center, Saskatoon, SK 5882, Canada; 3 Radiology & Nuclear Medicine, Sultan Qaboos Comprehensive Cancer Care and Research Centre (SQCCCRC), Muscat 112, Oman; 4 Research Laboratories Department, Sultan Qaboos Comprehensive Cancer Care and Research Centre (SQCCCRC), Muscat 112, Oman; 5 Medical Physics, Sultan Qaboos Comprehensive Cancer Care and Research Centre (SQCCCRC), Muscat 112, Oman; 6 Radiation Oncology, Sultan Qaboos Comprehensive Cancer Care and Research Centre (SQCCCRC), Muscat 112, Oman; 7 Medical Oncology, Sultan Qaboos Comprehensive Cancer Care and Research Centre (SQCCCRC), Muscat 112, Oman; 8 Medical School, Medical University of Lublin, 20-059 Lublin, Poland

**Keywords:** prostate cancer, oligometastatic disease, pelvic lymph node metastases, PSMA PET-CT, metastatic directed therapy, radiotherapy, androgen deprivation therapy (ADT), androgen receptor pathway inhibitors (ARPI)

## Abstract

**Background/Objectives**: Metastatic prostate cancer is increasing in the Gulf Cooperation Council countries. This study presents a multimodal treatment protocol incorporating radiotherapy to primary and metastatic sites, guided by PSMA PET/CT, combined with systemic therapy for non-metastatic pelvic node-positive and de novo low-volume metastatic prostate cancer. **Methods:** We conducted a retrospective cohort study of patients treated with radical radiotherapy doses (68 Gy/25 Fr or 78 Gy/39 Fr) to the prostate gland and gross pelvic disease, and SBRT (35–40 Gy/5 Fr) to distant bone metastases. All patients received LHRH agonists ± abiraterone/prednisone or enzalutamide. **Results:** Twenty-four consecutive patients were analyzed. The median age was 70.1 years (IQR, 65.7–77.7), the median baseline PSA was 27.9 ng/mL (IQR = 19.7–53.8), and median follow up was 24 months (IQR = 20.4–31.2). Clinical staging was cT3b in (46%), cT2 in (25%), cT4 in (17%), cT3a in (13%) of patients. Pelvic nodal involvement (cN1) was present in 91.7% of patients, while 54.1% had metastatic disease. Treatment was well tolerated. Acute toxicity was predominantly grade 1 genitourinary (GU) toxicity, occurring in 87.5% of patients, with grade 2 GU toxicity observed in 8.2% and no acute gastrointestinal (GI) toxicity. Late toxicity remained minimal, with grade 1 and grade 2 GU toxicity reported in 45.8% and 4.2% of patients, respectively, and no late GI toxicity. Mild systemic treatment-related toxicities were reported in 25% of patients, including sexual dysfunction, hypokalemia, muscle weakness, osteoporosis and depression/anxiety. At the six-month follow-up PSMA PET/CT assessment, 85.7% achieved a complete metabolic response, and 14.2% achieved a partial response. Biochemically, 75% of patients achieved undetectable PSA levels (<0.01 ng/mL), with all patients achieving a PSA nadir < 0.2 ng/mL. **Conclusions:** This first, hypothesis-generating real-world experience from the GCC, suggests that an integrated approach combining systemic therapy with metastasis-directed therapy is feasible. Prospective randomized studies are required to validate these results.

## Simple Summary

This study evaluated a combined treatment approach for patients with advanced prostate cancer in the Gulf region. Patients received hormone therapy together with targeted radiation treatment to the prostate, affected lymph nodes, and limited metastatic sites identified on advanced PSMA PET/CT scan. Most patients showed an excellent response, with major reductions in PSA levels and strong imaging responses, while treatment side effects were generally mild. These findings suggest that combining modern imaging, radiotherapy, and systemic treatment may offer a promising treatment strategy for selected patients with low-volume metastatic and node-positive non-metastatic prostate cancer.

## 1. Introduction

Prostate cancer is the second most common cancer among men worldwide. Approximately 1.46 million new cases were diagnosed in 2022 [1]. A higher incidence of prostate cancer was observed in developed countries of North America and Europe, largely attributed to increased awareness and access to care [2]. In contrast, metastatic disease at presentation remains relatively more prevalent in developing countries, likely due to delayed diagnosis, limited screening, social barriers, and constrained healthcare resources [3,4].

In recent years, the Gulf Cooperation Council (GCC) countries have seen a rise in prostate cancer incidence, which is partially due to aging populations, lifestyle changes, and increased awareness and screening [4]. Age-standardized incidence rates of 5–10 per 100,000 have been reported in some populations within the region, with a substantial proportion of patients still presenting with advanced or metastatic disease [5]. Despite advances in therapies, heterogeneity in trial designs, disease burden definitions, and treatment strategies has limited consensus regarding the optimal multimodal treatment approach for low-volume metastatic prostate cancer [6]. Current management of de novo low-volume metastatic disease commonly includes androgen deprivation therapy (ADT) combined with androgen receptor pathway inhibitors (ARPIs), while metastasis-directed therapy (MDT) using stereotactic body radiation therapy (SBRT) is increasingly being explored in carefully selected patients with limited disease volume [7,8].

However, there remains a lack of regional consensus in the GCC regarding standardized treatment protocols for metastatic prostate cancer. In this context, we report early real-world outcomes from a single-center cohort in Oman, comprising patients with pelvic node-positive non-metastatic (cN1 cM0) and de novo low-volume metastatic prostate cancer treated with systemic therapy in combination with radical radiotherapy. These findings are hypothesis-generating and represent early institutional experience.

## 2. Materials and Methods

### 2.1. Study Design

An observational retrospective cohort study was conducted at the largest tertiary oncology referral center. The study was approved by the institutional research ethics committee (38-2022). A consecutive series of 24 male patients with regional node-positive non-metastatic (cN1 cM0) or de novo metastatic low-volume prostate cancer, irrespective of age, treated between August 2021 and October 2023, were included in the study. No eligible patients treated during the study period were excluded from analysis.

### 2.2. Patient Staging and Treatment Protocol

Baseline staging was performed using CT and/or MRI in combination with PSMA PET/CT scans. Final clinical staging was assigned following multidisciplinary discussion within the genitourinary (GU) tumor board, integrating the PSMA PET/CT findings with conventional imaging and clinical parameters [9]. All PSMA PET/CT interpretations were jointly reviewed by a dedicated nuclear medicine physician and radiologist to minimize the risk of false-positive and false-negative interpretations.

All target volumes were delineated using registered CT simulation and PSMA PET/CT images. For definitive prostate radiotherapy, the clinical target volume (CTV) includes the prostate gland with or without the seminal vesicle (SV) according to disease extent. An isotropic 5 mm margin was applied to create the Planning Target Volume (PTV). Grossly involved nodes were countered and individually expanded by 5 mm to create the PTV. Uninvolved lymph nodes and where require uninvolved Seminal Vesicles were countered as the CTV and expanded by 5 mm to create PTV. The distant metastatic GTV was expanded by a uniform 5 mm margin to create the PTV. 

**Figure 1:**
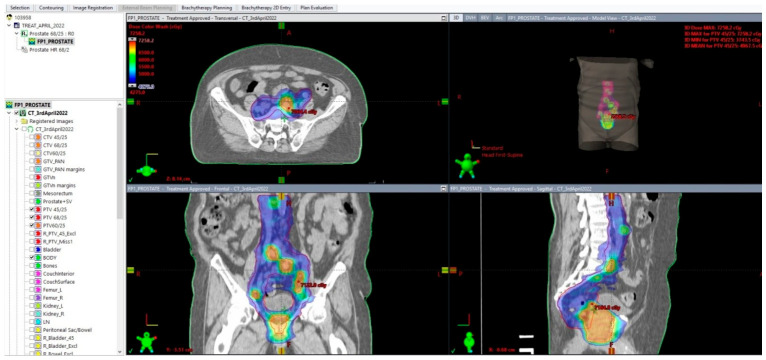
Figure shows targets ( PTV 45, PTV 60 and PTV 68) and dose clouds (Blue: 45 Gy, green: 60 Gy and orange: 68 Gy). (a) Single axial pelvic image at the level of pelvic bones. (b) Single coronal abdomen and pelvis images. (c) Single sagittal abdomen and pelvis images. (d) Surface view.

A total of 22 patients received moderately hypofractionated radiotherapy (68 Gy in 25 daily fractions), while 2 patients received conventional radiotherapy (78 Gy in 39 daily fractions) to the gross pelvic disease (prostate ± seminal vesicle ± gross pelvic nodes). Dose fractionation schedules were decided based on the physicians’ preference. Para-aortic grossly involved nodes, when present, were treated with 60 Gy in 25 fractions concurrently. Elective nodal irradiation to the pelvis and/or para-aortic area, if required, was given at doses of 45–50 Gy in 25 fractions concurrently (Figure 1). VMAT treatment planning was utilized to generate plans. Patients with distant bone metastases outside the abdomen and pelvis received SBRT (35–40 Gy) in 5 consecutive fractions during the main course of treatment. CBCT was used to deliver image-guided radiotherapy (IGRT).

ADT consisted of subcutaneous injections of goserelin 10.8 mg administered every three months, starting in the neoadjuvant phase, continued concurrently with radiotherapy, and maintained in the adjuvant setting. Systemic Therapy was further intensified with either abiraterone acetate (1000 mg daily) and prednisone (5 mg daily) or enzalutamide (160 mg daily) and continued for up to 2 years. Apalutamide was not available at our institution during the study period. Moderate hypofractionation of 68 Gy in 25 fractions is considered biologically equivalent to standard doses of 78 Gy in 39 fractions [10].

### 2.3. Data Collection and Analysis

Clinical, demographic, radiotherapy planning parameters, treatment delivery details, toxicity profiles, and oncological outcomes were systematically collected and recorded in a password-protected database accessible only to study investigators.

Baseline and follow-up PSMA PET/CT imaging was performed at 6–9 months post-treatment. Response assessment was conducted using the Response Evaluation Criteria in PSMA PET/CT (RECIP 1.0), classifying the metabolic response as complete response (CR), partial response (PR), stable disease (SD), and progressive disease (PD) based on changes in PSMA-avid disease [11]. It should be noted that RECIP 1.0 criteria were originally developed for metastatic castrate-resistant prostate cancer, and their applicability in de novo low-volume metastatic and node-positive non-metastatic disease remains under investigation; therefore, the results should be interpreted with caution in these settings.

Biochemical response was evaluated using serial PSA levels, which were collected at every follow-up visit. Treatment-related toxicities were assessed retrospectively through a review of patients’ clinical records and graded according to CTCAE v5.0. Acute toxicities were events occurring within 90 days of the radiotherapy completion date.

Descriptive statistics were used to summarize the data. Analysis was performed using median, interquartile range (IQR), mean, and standard deviation for continuous variables analysis, and frequencies and proportions for categorical variables analysis [12]. All analyses were performed using the R statistical software version 4.4.1 [13].

## 3. Results

Twenty-four consecutive patients were included in the study (Table 1). The median age at diagnosis was 70.1 years (Interquartile range [IQR], 65.7–77.7), with a median follow-up duration of 24 months (IQR, 20.4–31.2). The median baseline PSA level was 27.9 ng/mL (IQR, 19.7–53.8).

At presentation, most patients (75%) had locally advanced primary disease (cT3-4). The T stage distribution was as follows: cT2 in 25% (6/24), cT3a in 12.5% (3/24), cT3b in 45.8% (11/24), and cT4 in 16.7% (4/24). Pelvic nodal involvement (cN1) was present in 91.7% (22/24) of patients, with a median of 3.5 involved pelvic lymph nodes per patient (IQR, 3–7.75) and a median nodal size of 1 cm (IQR, 1.0–1.9 cm). Para-aortic nodal involvement (M1a) was identified in 37.5% (9/24) of patients, with a median of 4 involved para-aortic lymph nodes (IQR, 2.2–5.0). Bone metastases (cM1b) were present in four patients (16.7%), involving the left pubic ramus, iliac bone, ribs, and humerus.

Germline genetic testing was offered to all patients; however, only 37.5% (9/24) of patients consented to testing. Pathogenic BRCA mutations were identified in 22.2% (2/9) of the tested patients, while no clinically significant variants were detected in the remaining seven patients [14].

All patients received ADT and radiation therapy (Table 2). Most patients (21/24, 87.5%%) continued hormonal therapy, whereas it was discontinued in three (12.5%) patients after achieving remission following a shared decision-making process with their treating physician regarding the risks and benefits of continuation vs. discontinuation. Treatment intensification with ARPIs was administered in 79.2% (19/24) of patients, including abiraterone acetate plus prednisone or enzalutamide.

Acute treatment-related toxicity was predominantly mild genitourinary (GU), with grade 1 toxicity observed in 87.5% (21/24) and grade 2 toxicity in 8.2% (2/24) of patients. No acute gastrointestinal (GI) toxicity was observed. During the late follow-up (from six months onwards), grade 1 GU toxicity was reported to be 45.8% (11/24), and grade 2 GU toxicity in was reported in one (4.2%) patient, with no late GI toxicity recorded. Hormonal therapy-related side effects were reported in 25% of patients and included sexual dysfunction, hypokalemia, muscle weakness, osteoporosis, and depression/anxiety (Table 3).

A follow-up PSMA PET/CT scan was performed in 21/24 patients at 6–9 months post-treatment. Repeating the PMA PET/CA scan was not a mandatory component of follow-up at our center; therefore, not all patients underwent repeat imaging. The absence of post-treatment imaging in three patients was unrelated to disease or treatment.

**Figure 2:**
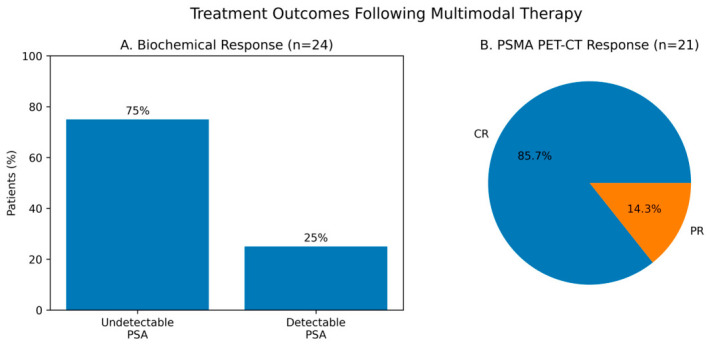
Treatment outcomes following multimodal therapy. (**A**) Biochemical response at last follow-up. Eighteen of 24 patients (75%) achieved an undetectable PSA level (<0.01 ng/mL), while six patients (25%) had detectable PSA levels ranging from 0.02 to 0.19 ng/mL. (**B**) Metabolic response on follow-up PSMA PET/CT performed 6–9 months after treatment. Among 21 evaluable patients, complete metabolic response (CR) was observed in 18 patients (85.7%) and partial metabolic response (PR) in 3 patients (14.3%).

Among the 21 patients who underwent repeat imaging, 85.7% (18/21) achieved a complete response (CR) with a 95% confidence interval = 63.7–97.0%, while 14.3% (3/21) achieved a partial response (PR) (Figure 2). To assess the robustness of these findings, a sensitivity analysis was performed for the full cohort (*n* = 24). In the worst-case scenario, assuming that all three non-evaluable patients had progressive disease, the overall CR rate would be 75% (18/24). In the best-case scenario, assuming CR in all three patients, the CR rate would be 87.5% (21/24). This analysis showed that the overall interpretation of the high metabolic response remains unchanged. Biochemically, 75% (18/24) of the entire cohort achieved undetectable PSA levels (<0.01 ng/mL), with a 95% confidence interval of 53.3–90.2%, and the highest PSA recorded during the follow-up was 0.19 ng/mL (Figure 2).

Both patients with BRCA mutations achieved a complete metabolic response on PSMA PET/CT; one had an undetectable PSA level, while the other had detectable but low PSA (≥0.02 ng/mL). Among the seven patients without clinically significant genetic variants, 42.9% achieved a complete metabolic response, and 57.1% achieved a PR, with four achieving undetectable PSA. Given the limited genetic testing data, these findings should be considered hypothesis-generating rather than clinically significant.

## 4. Discussion

In this retrospective single-institution study, we evaluated the outcome of an integrated multimodal treatment strategy, combining systemic therapy intensification, definitive radiotherapy to the primary tumor, and MDT in patients with regional node-positive non-metastatic (cN1 cM0) and de novo low-volume metastatic prostate cancer. This multimodal treatment was well tolerated, supporting the safety and feasibility of this treatment-intensified approach. Predominant radiotherapy-related toxicity was low-grade GU toxicity, with no GI toxicity. Systemic treatment-related adverse events were manageable and consistent with the expected safety of ADT and ARPI intensification. However, adverse events may have been underreported due to the retrospective nature of the study and reliance on medical record documentation. With a median follow-up of 24 months, this approach was associated with encouraging disease control, with three out of four patients achieving undetectable PSA levels and all patients maintaining PSA levels less <0.2 ng/mL during follow-up. Furthermore, response assessment using follow-up PSMA PET/CT demonstrated a high rate of metabolic response, with 85.7% of evaluated patients achieving CR. These findings should, however, be interpreted in the context of a retrospective design, a small cohort size, a relatively short follow-up, and heterogeneity of the study population.

For patients with regional node-positive non-metastatic disease (cN1 cM0), contemporary guideline frameworks, including the European Association of Urology, support an aggressive locoregional treatment strategy incorporating long-term ADT and definitive radiotherapy to the prostate and pelvic lymph nodes. This treatment paradigm formed the basis of management of our cN1 cM0 cohort and reflects an evolving concept that pelvic node-positive disease represents a biologically advanced but potentially curable state [15].

The role of prostate-directed therapy in low-volume metastatic hormone-sensitive prostate cancer has been established by the STAMPEDE trial (arm H), which demonstrated improved overall survival in patients with low metastatic burden, while no survival benefit was observed in the unselected metastatic population. Long-term follow-up confirms the durability of this benefit [16,17]. These findings are further reinforced by the STOPCAP meta-analysis, which showed that the benefit of prostate radiotherapy is largely confined to patients with low-volume disease. Unlike STAMPEDE, where radiotherapy was directed only to the primary tumor, our institutional strategy incorporated both definitive prostate radiotherapy and MDT to metastatic sites, reflecting a broader multimodal intensification approach [18]. However, given the retrospective design, lack of a control group, and differences in patient selection and treatment delivery, our findings should be interpreted cautiously.

Systemic treatment intensification with ARPIs in combination with ADT has become an established standard in metastatic hormone-sensitive prostate cancer. Trials such as ENZAMET demonstrated improved OS with enzalutamide, while the PEACE-1 trial strengthened the evidence for upfront treatment intensification with abiraterone-based strategies [19,20,21]. In our cohort, ARPI intensification was incorporated in the majority of patients (79%) as part of the overall multimodal treatment strategy, reflecting contemporary evidence-based systemic escalation.

The role of MDT in oligometastatic prostate cancer continues to evolve. The ORIOLE trial demonstrated improved progression control with SBRT compared with observation, supporting the biological rationale for ablative treatment of limited metastatic disease [22]. More recently, the EXTEND trial demonstrated improved PFS when comprehensive MDT was added to standard systemic therapy, aligning more closely with the integrated strategy used in our cohort [23]. The broader concept of ablative treatment for oligometastatic disease is further supported by the SABR-COMET trial, which is expected to improve survival across multiple types [24].

While prospective studies such as ORIOLE, STOMP, and EXTEND have demonstrated improvements in disease control with MDT, direct comparison with our cohort should be interpreted cautiously because of important differences in study design and treatment strategies. Most prospective MDT trials enrolled patients with recurrent oligometastatic disease and primarily evaluated progression-related endpoints. In contrast, our cohort consisted of patients with newly diagnosed regional node-positive non-metastatic and de novo low-volume metastatic disease treated with a comprehensive multimodal approach that combined definitive treatment of the primary tumor, treatment of involved nodes and metastatic sites, and systemic treatment intensification. Nevertheless, the favorable biochemical and imaging outcomes observed in our study are consistent with the emerging concept that MDT may improve disease control in carefully selected patients with limited metastatic burden. The low incidence of clinically significant toxicity observed in our cohort further supports the feasibility of integrated PMA PET-guided MDT within a broader treatment intensification strategy.

An important distinguishing feature of our study is the incorporation of PSMA PET/CT into both staging and response assessment. Compared with conventional imaging, PSMA PET/CT provides improved sensitivity for nodal and distant metastatic disease, enabling more precise staging and better selection for MDT [25]. This may have contributed to the high metabolic response rates observed in our cohort. However, the improved sensitivity of PSMA PET/CT may also introduce stage migration and possible overestimation of the disease volume. To minimize this risk, all imaging findings were reviewed in a multidisciplinary GU tumor board with dedicated nuclear medicine and radiology input.

Our study has several limitations. First, the retrospective single-center design introduces inherent selection bias and limits the ability to establish causal relationships between treatment and outcomes. Second, the relatively small sample size (*n* = 24) reduces statistical power and limits generalizability. Third, the absence of a control group precludes direct comparison with systemic therapy alone and limits conclusions regarding the incremental benefits of the multimodal treatment strategy. Furthermore, the inclusion of both regional node-positive non-metastatic (cN1 cM0) and the de novo low-volume metastatic (cM1) patients reflects real-world practice but introduces clinical heterogeneity. In addition, although the median follow-up duration of 24 months allowed assessment of early biochemical and metabolic responses, it remains insufficient to evaluate long-term PFS, OS, and late treatment-related toxicities. A longer follow-up is needed to determine the durability of these findings. Finally, the use of RECIP 1.0 criteria for metabolic response assessment should also be interpreted cautiously, as their utility in de novo low-volume metastatic and regional node-positive non-metastatic disease has not yet been fully validated. Consequently, these findings should be considered hypothesis-generating and require confirmation in prospective multicenter studies with larger cohorts, appropriate control groups, and longer follow-up.

Despite these limitations, this study provides early real-world evidence supporting the feasibility of an integrated treatment strategy combining systemic intensification, definitive local Radiotherapy, and PSMA PET/CT-guided metastatic-directed therapy in carefully selected patients with node-positive non-metastatic and de novo low-volume metastatic prostate cancer. Prospective multicenter studies with longer follow-up are needed to validate these findings and determine whether this approach translates into a durable survival benefit.

## 5. Conclusions

This real-world single-institution study suggests that an integrated multimodal strategy combining systemic intensification, definitive prostate radiotherapy, and PSMA PET/CT-guided metastasis-directed therapy is feasible, well-tolerated, and associated with encouraging early biochemical and metabolic responses in patients with node-positive non-metastatic and de novo low-volume metastatic prostate cancer. While these findings support the potential role of treatment intensification in carefully selected patients, the retrospective design, limited cohort size, and relatively short follow-up preclude a definitive conclusion. Prospective multicenter studies with longer follow-up are warranted to validate these outcomes and define the long-term survival benefit of this approach.

## Figures and Tables

**Table 1:** Characteristics of the study population (*N* = 24).

Variable	Category	Value
Age (Y)	Min/Max	48.3/96.4
	Median [IQR]	70.1 [65.7; 77.7]
	Mean (Standard Deviation)	70.9 (10.4)
	Number	24
Follow-up Time (months)	Min/Max	12/53
	Median [IQR]	24 [20.4; 31.2]
	Mean (Standard Deviation)	26.4 (10.8)
	Number	24
PSA ng/mL @diagnosis	Min/Max	13.0/200.0
	Median [IQR]	27.9 [19.7; 53.8]
	Mean (Standard Deviation)	51.5 (52.8)
TNM	Number	24
T stage	T2	6/24 (25.0%)
	T3a	3/24 (12.5%)
	T3b	11/24 (45.8%)
	T4	4/24 (16.7%)
N stage (Pelvic node)	N0	2/24 (8.3%)
	N1	22/24 (91.7%)
M stage	M0	11/24 (45.8%)
	M1	13/24 (54.1)
	M1a	9/13 (69.2%)
	M1b	4/13 (30.7%)
PA node	No	15/24 (62.5%)
	Yes	9/24 (37.5%)
Largest node	Min/Max	0.5/6.9
	Median [IQR]	1.0 [1.0; 1.9]
	Mean (Standard Deviation)	1.5 (1.3)
	Number	22
Genetic Variants	BRCA2	2/9 (22.2%)
	No Variant of Clinical Significance Identified	7/9 (77.8%)

**Table 2:** Treatment and outcomes.

Variable	Category	Value
Continued therapy	No	3/24 (12.5%)
	Yes	21/24 (87.5%)
Dual hormonal therapy	No	5/24 (20.8%)
	Yes	19/24 (79.2%)
PET response	CR	18/21 (85.7%)
	PR	3/21 (14.2%)
PSA response	Detectable ≤ 0.02 to 0.19 ng/mL	6/24 (25.0%)
	Undetectable < 0.01 ng/mL	18/24 (75.0%)
	Nadir < 0.2 ng/mL	24/24 (100%)

**Table 3:** Treatment-related adverse events.

Variable	Category	Value
GI toxicity (0–3 months after starting therapy)	No	24/24 (100.0%)
	Yes	0/24 (0%)
GU toxicity (6 months to last follow-up)	None	12/24 (50.0%)
	Grade 1	11/24 (45.8%)
	Grade 2	1/24 (4.2%)
GI toxicity (6 months to last follow-up)	No	24/24 (100.0%)
	Yes	0/24 (0%)
Hormonal Therapy-related adverse events		
	No	18/24 (75%)
	Yes	6/24 (25%)

## Data Availability

The datasets generated and/or analyzed during the current study are not publicly available due to institutional privacy regulations and patient confidentiality requirements.

## Abbreviations

The following abbreviations are used in this manuscript:

GCC	Gulf Cooperation Council
ADT	Androgen Deprivation Therapy
LHRH	Luteinizing Hormone Releasing Hormone
ARPI	Androgen Receptor Pathway Inhibitor
MDT	Metastasis Directed Therapy
SBRT	Stereotactic Body Radiotherapy
PSMA	Prostate-Specific Membrane Antigen
PET-CT	Positron Emission Tomography—Computed Tomography
MRI	Magnetic Resonance Imaging
RECIP	Response Evaluation Criteria in PSMA Imaging
CR	Complete Response
PR	Partial Response
SD	Stable Disease
PD	Progressive Disease
CTCAE	Common Toxicity Criteria for Adverse Events
PSA	Prostate Specific Antigen
IQR	Interquartile Range
BRCA	Breast Cancer Gene
GU	Genito-urinary
GI	Gatro-intestinal
